# Informal employment and high burden of out-of-pocket healthcare payments among older workers: evidence from the Longitudinal Ageing Study in India

**DOI:** 10.1093/heapol/czae074

**Published:** 2024-08-03

**Authors:** Poulomi Chowdhury, Srinivas Goli

**Affiliations:** Health Research Institute, Faculty of Health, University of Canberra, Bruce, Canberra, ACT 2617, Australia; International Institute for Population Sciences, Deonar, Mumbai, Maharashtra 400088, India

**Keywords:** Informal workers, older population, healthcare, out-of-pocket (OOP) payments, India

## Abstract

India’s economy is among the fastest growing in the world. However, a large share of informal workforce is a common characteristic of country’s economy, comprises a significant portion of most of its labour markets. This workforce often receives low wages and lacks benefits such as strong social security and health coverage for all. The majority of healthcare spending in India is private. As India’s population ages and the informal sector expands, it is expected that many of these workers will continue to work beyond the retirement age to bear their own healthcare costs due to lack of savings, pensions, and the precarious nature of their employment. In this context, this study estimates the burden of out-of-pocket (OOP) payments on India’s informal older workers compared to their formal counterparts, using data from the first wave of the nationally representative Longitudinal Ageing Study in India. According to estimates from the two-part regression model, informal older workers pay, on average, INR 1113 (*P* < 0.01) and INR 55 (*P* < 0.05) less than their formal counterparts for inpatient and outpatient care, respectively. Further, probit regression models revealed that the burden of combined (both inpatient and outpatient) OOP payments exceeding (by 40, 20 and 10%) of their income is significantly higher among informal older workers compared to formal older workers. The study underscores the need for strengthening of universal health insurance schemes to ensure everyone has access to medical services without experiencing financial hardship. It also advocates for policies specifically tailored towards informal workers, considering their unique challenges with regard to livelihoods and healthcare security. In particular, this encompasses bolstering the existing social security and healthcare system, and related policies for ensuring financial security against OOP payments, especially for informal workers and all the population in general.

Key messages

- The study provides a key insight into the burden of OOP healthcare payments among older workers using nationally representative data of the Longitudinal Ageing Study in India, a unique survey that collects information on the health, economic, work, retirement and social aspects of older people in India.
- The OOP payments among informal older workers are significantly lower than those of formal older workers.
- Informal older workers suffer more from the burden of catastrophic healthcare payments than their formal counterparts.
- Older workers afflicted with either CHC or a combination of CHC and depression are prone to the higher burden of OOP payments.

## Introduction

Households that do get needed care do not suffer undue financial hardship as a result of healthcare payments, referred in health economics literature as ‘financial protection’ (Wagstaff *et al*., 2018). Financial protection is directly impacted by health financing policies. Therefore, one of the ultimate coverage goals and the cornerstone of Universal Health Coverage is financial security. Sustainable Development Goal 3.8.2 also emphasizes the national targets for reducing catastrophic healthcare expenditure (CHE). Evidence suggests that financial hardship is caused by poor financial protection mechanisms for health, which result in out-of-pocket (OOP) medical payments and financial barriers to getting healthcare (Wagstaff *et al*., 2018). Thus, countries strive to establish healthcare systems capable of delivering affordable, high-quality and easily accessible healthcare services.

However, for low- and middle-income countries, providing such quality healthcare services presents a significant challenge, with OOP payments being the primary means of healthcare financing (Sangar *et al*., 2020; Shrank *et al*., 2021; Prinja *et al*., 2022). An illustration of this challenge is evident in a study across 15 African nations, showcasing the impact of OOP healthcare expenditure, ranging from 23% of households in Zambia to a staggering 86% in Burkina Faso (Leive and Xu, 2008). Global statistics in 2017 revealed that 996 million people faced catastrophic health spending, with 70 million falling into poverty due to the high burden of healthcare expenditures. The second largest number is seen in Asia, next only to Latin America, where most people who are facing catastrophic payments (WHO 2021, 2021).

In India, a parallel narrative unfolds as households bear a substantial financial burden for medical treatment, with approximately 65% of healthcare expenses being incurred through OOP payments at the point of service delivery — ranking India among the countries with one of the highest OOP expenditures globally (Reddy *et al*., 2011; Pandey *et al*., 2018b; National Health System Resource, 2019; Prinja *et al*., 2022). Additionally, 49% of Indian households seeking hospitalization or outpatient care encounter CHE, leading to impoverishment for 15% of these households (Nanda and Sharma, 2023). Other estimates suggest that 39 to 50 million households in India fall below the poverty line annually due to high OOP healthcare payments (Hooda, 2017; Selvaraj *et al*., 2018). Over the past decade, OOP outpatient expenditure in India has surged by over 100%, while the cost of inpatient care has risen by almost 300% (Jayakrishnan *et al*., 2016; Pandey *et al*., 2018a).

Furthermore, it is observed that CHE are more likely to occur during old ages compared to younger age groups across all geographies and socio-economic groups (Mohanty *et al*., 2014; 2016; Baird, 2016; Zhai *et al*., 2017; Carreras *et al*., 2018; Pandey *et al*., 2018a; Panda and Mohanty, 2022). Prior research indicates that OOP healthcare expenditure for inpatient care is two times higher for the older population compared to their younger counterparts (Kastor *et al*., 2018; Mondal and Dubey, 2020). Estimates suggest that over half of the older population is affected by CHC, with a quarter suffering from multi-morbidity (Agrawal *et al*., 2014; Talukdar and Himanshu, 2017). The staggering healthcare expenditure among older individuals reflects their deteriorating health condition in later life caused by chronic diseases and multi-morbidity (Schoenberg *et al*., 2007; Srivastava *et al*., 2022). Moreover, the poor health conditions coupled with an over-reliance on private healthcare facilities lead to financial catastrophe among the older population (Brinda *et al*., 2015; Tiagi, 2016; Jeyashree *et al*., 2018; Pandey *et al*., 2018a; Panda and Mohanty, 2022).

Medicines are considered to be the single most important factor of the OOP payments, accounting for approximately 70% of total OOP payments in India (Prinja *et al*., 2022). Additionally, the growing reliance on private healthcare, compounded by the absence of adequate medical and social insurances and escalating medical care costs, remain a principal cause of direct debt and poverty in India (Balarajan *et al*., 2011). Despite this alarming scenario, the government health expenditure in India remains dismally low at 1.15% of the gross domestic product (Government of India, 2017; Nanda and Sharma, 2023).

The labour market of any economy is typically divided into formal and informal segments. Developing countries, in particular, have an overwhelming number of informal workers, estimated to be around 900 million (Mishra, 2017). The International Labour Organization (ILO) defines informal employment as both self-employment and wage employment that is not registered, regulated or protected by existing legal or regulatory frameworks (ILO, 2015). Informal workers also lack secure employment contracts, worker benefits, social protection and representation. This description of informal workers is particularly applicable to India (ILO, 2015). The recent data indicate that about 92.4% of the workforce is engaged in informal work (GOI, 2019; Ramana Murthy, 2019), largely due to the high levels of rural and unorganized sectors of the economy, poverty and unemployment in India (Mishra, 2017; Rains and Wibbels, 2023). However, it is important to note that while poverty and unemployment are common among informal workers in India, they are not definitive characteristics of this group but rather key features of the informal economy. With an increasing older population, high cost of living and lack of access to old-age security in India, many older individuals continue to work past the retirement age of 60 years (Rajan, 2010; Reddy, 2016; Reddy and Goli, 2023; Chowdhury *et al*., 2023).

Previous research has highlighted that continuing to work into old age is primarily a survival strategy in countries, where a significant portion of employment is in informal sectors lacking adequate social safety nets (Reddy, 2016; Reddy and Goli, 2023). This is evident in India, where the 2011 Census reported that approximately 33 million people are working beyond retirement age, with the majority engaged in the informal sector (Census of India, 2011). A joint study by the Secretariat of the World Trade Organization and the International Labour Office (2009) reveals that the informal sector is the primary source of livelihood when social security provisions, such as unemployment insurance, financial assistance and pensions are minimal (Mishra, 2017; Rains and Wibbels, 2023). In contrast, the central and state government employees, along with a small portion of the formal workforce, are covered by social protection schemes (Dandona *et al*., 2017; Khan, 2021). Additionally, the Employees Provident Fund serves as a major benefit for the formal workforce, providing post-retirement financial support (Sakthivel and Pinaki, 2006; Khan, 2021). However, there is a significant wage gap, social and health care protection provisions between these two groups, resulting in a greater capacity to pay for health among formal workers (Kumar and Ranjan, 2015; Kumar and Pandey, 2021).

To provide financial aid to Indian workers in the informal sector, initiatives like the Janashree Bima Yojana (2000) and the Universal Health Insurance Scheme (2003) were launched (Government of India, 2020; Srivastava *et al*., 2023). Despite these efforts, numerous implementation challenges led to the establishment of the Unorganised Workers Social Security Act, which also fell short of providing adequate social security (Mishra, 2017; Srivastava *et al*., 2023). Several nationwide programs aimed at the older population, such as the National Policy on Older Persons and the National Social Assistance Program, failed to deliver sufficient financial support, especially to those in the informal sector and below the poverty line (Gokhale, 2003; Bharati and Singh, 2013; Dhillon and Ladusingh, 2013). Goli *et al*. (2019) highlights issues such as insufficient coverage, inadequate benefits, lack of awareness, irregular payments and corruption.

The Rastriya Swasthya Bima Yojana (RSBY) aimed to address these shortcomings but encountered similar issues, failing to adequately cover vulnerable populations adequately (Narayana, 2010; Sun, 2011; Ghosh, 2014). Poor program performance has been a primary reason for the lack of financial and social protection among older people in India, exacerbating their vulnerability due to escalating healthcare costs and low insurance coverage (Carreras *et al*., 2018; Jeyashree *et al*., 2018; Lyons *et al*., 2018; Agrawal and Mishra, 2021; Sahoo *et al*., 2021; Mohanty *et al*., 2023; Panda and Mohanty, 2022). The government of India launched the Ayushman Bharat Program (ABP) as part of its commitment to Universal Health Coverage. The ABP aims to enhance the accessibility, availability and affordability of primary, secondary and tertiary healthcare services across India (National Health Authority, 2018). However, similar to the earlier RSBY, which floundered due to its flawed insurance-based approach, Ayushman Bharat also depends on private health providers (EPW Engage, 2020; Sarvesetty, 2023). Despite its noble objectives, the program has encountered significant challenges, including corruption, lack of awareness and insufficient participation (Agrawal and Mishra, 2021; Sarvesetty, 2023).

According to a United Nations (2019), the older population (60 years and above) in India is projected to reach 319 million by 2050 (United Nations, 2019). With the increasing share of informal sector employment in India (Ramana Murthy, 2019), it is likely the proportion of older people in this sector will also rise due to the lack of adequate financial and social protection schemes. A recent study has spotlighted that both informal and formal older workers in India suffer from various adverse health outcomes (Chowdhury *et al*., 2023). This, coupled with the socio-economic disparities between formal and informal older workers, raises two critical questions: (1) What is the extent of OOP healthcare expenditure among formal and informal older workers? (2) Does the burden of OOP healthcare expenditure vary by type of work? To date, no study has addressed these research questions. Therefore, this study aims to examine the vulnerable segment of the older population in India. By focusing on the vulnerable segment of the population, this study provides a robust foundation for policy interventions designed to reduce healthcare inequities and enhance social protection. Further, this research can pave the way for further studies examining healthcare expenditures and social protection mechanisms, especially in other low- and middle-income countries facing similar challenges.

## Materials and methods

### Data source

**Figure 1.**
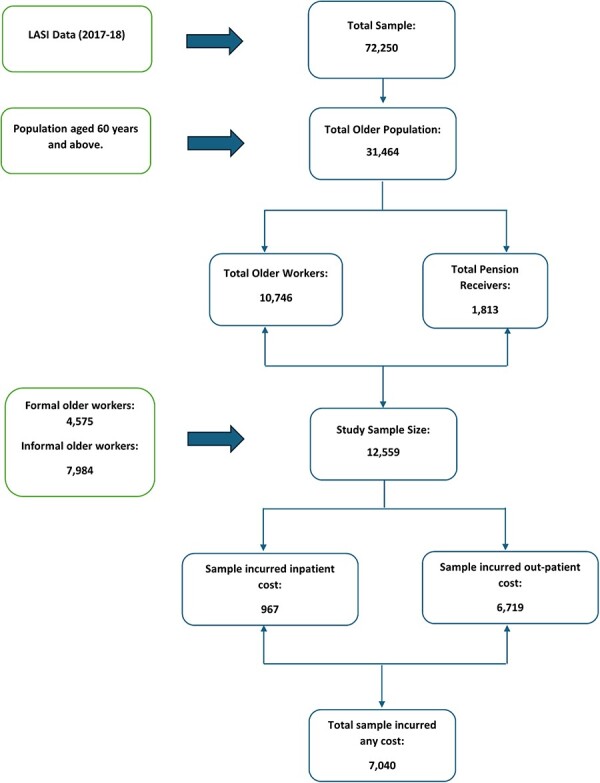
Flow chart of sample size

This paper uses data from the Longitudinal Ageing Study in India (LASI), a cross-sectional survey that covers the population aged 45 years and above in India. The survey was conducted in 2017–18 by the International Institute for Population Sciences, in collaboration with Harvard TH Chan School of Public Health and the University of Southern California (IIPS, NPHCE, MoHFW, HSPH & USC, 2020). The survey used a multistage-stratified cluster sampling design to collect data from 72 250 Indian adults aged 45 years and above. The data include information on social and economic well-being, functional health, disease burden, healthcare utilization, healthcare costs and childhood health conditions (IIPS, NPHCE, MoHFW, HSPH & USC, 2020). The data also cover the working population across ages and sectors, work conditions, health insurance, pensions and post work benefits. Out of a total sample of 72 250 individuals, 31 464 belong to the age group of 60 years and above. For this study, a sample size of 12 559 older individuals (aged 60 years and above) was selected. This includes those who are currently working (*n* = 10 746) or receiving a pension (*n* = 1813). Among the selected sample, 7040 individuals have incurred any healthcare costs. Details of the sample size information are provided in the Figure 1.

### Outcome variables

The main outcome variable of the study is the OOP payments for healthcare services. The study considers both inpatient and outpatient OOP costs. The OOP cost includes all the expenses for hospitalization or outpatient visits in the last 12 months. The expenses are health care provider’s fees, medicines from hospital or outside, tests or investigation, hospital and nursing charges including bed charges and food, operation theatre charges, surgery charges and related expenses, cost of blood and oxygen, transport and other expenses. The OOP cost is net of any reimbursement by employer or other agencies.

OOP payments only measure the cost of healthcare services, but not the burden on the individual. To measure the burden, the study calculates the ratio of OOP payments to the monthly income from wage or pension (for older people aged 60 years and above). We have estimated CHE.

Catastrophic payments headcount is given by the formula:


(1) $$CH{E_i} = \frac{1}{N}{\ }\mathop \sum \limits_{i = 1}^N (\frac{{OO{P_i}}}{{incom{e_i}{\ }}}) > Z\,\,\ldots\ldots\ldots\ldots.$$

Where, $CH{E_i}$ catastrophic payments for ith individual. *N* is the sample size. $CH{E_i}$ is defined when $\frac{{OO{P_i}}}{{incom{e_i}{\ }}}$ of ith individual is greater than a demarcated cut-off point threshold $Z$ (i.e. 10, 20 and 40% in this case). Theoretical minimum and maximum of values of catastrophic payment headcount are 0 and 100%, respectively.

### Exposure variable

The main exposure variable in this study is ‘type of work of the older workers aged 60 years and above’. The LASI survey uses the International Classification of Occupation, 2015 to classify the types of occupations. These types are further categorized into formal and informal work as per the 66th round of National Sample Survey Organization report (NSSO, 2004), following the National Classification of Occupation, 2004 (NCO, 2004). The type of work variable is thereby coded as 0 ‘formal’ or 1 ‘informal’. Moreover, the older individuals receiving pension after the retirement (*n* = 1813) are also included in the formal category. The categorization of the types of occupation into formal and informal is described in Chowdhury et al. (2023); Chowdhury and Singh (2024).

### Other covariates

The selection of other covariates in this study is based on previous research related to healthcare expenditure among older population (Mohanty *et al*., 2014; 2023; Brinda *et al*., 2015; Jeyashree *et al*., 2018; Pandey *et al*., 2018a; 2018b; Sahoo *et al*., 2021; Panda and Mohanty, 2022) and potential factors affecting later life work engagement of older workers (Adhikari *et al*., 2011; Robroek *et al*., 2013; Dantas *et al*., 2017; Lee and Kim, 2017; Vives *et al*., 2018; Chowdhury *et al*., 2023; Chowdhury and Singh, 2024). These factors are ordered in four major dimensions which are socio-economic and demographic, health, lifestyle behaviours and regions. These dimensions have been controlled in the subsequent models to study the main effect of type of work on healthcare expenditure of older workers. The socio-economic and demographic dimension includes gender (male, female), age groups (60–65, 65+), caste groups [general, scheduled tribe (ST), scheduled caste (SC), other backward class (OBC)], religion (Hindu, Muslim, others), education level (low, middle, high), marital status (currently married, others), residence (rural, urban), wealth (low, middle, high) and household size (1, 2, 3, 4+).

Health dimension includes health condition (no condition, CHC, depression, and CHC and depression), childhood health (good/fair, poor) and any health insurance (no, yes). The health condition variable in this study is the combination of CHC and depression. CHC is constructed using nine major self-reported chronic illnesses, such as chronic heart diseases, hypertension, cancer, diabetes, chronic lung disease, arthritis, stroke, high cholesterol and neurological problems. Here CHC is a binary variable where 0 includes no ailment and 1 includes at least one health condition. Further, the depression is constructed using self-reported scale of the Centre for Epidemiological Studies (CES-D). There are 10 indicators under this scale, which are combined for aggregate score ranging from 0 to 10. Participants with a score of four or more are considered to have depressive symptoms and coded as 1.

The other dimensions consist of variable such as smoking/consuming tobacco (no, yes), drinking alcohol (no, yes), vigorous activities (never, rare, everyday), moderate activities (never, rare, everyday), yoga (never, rare, everyday) and regions (north, central, east, northeast, west, south, union territories).

### Statistical analysis

The study estimates the descriptive statistics of inpatient and outpatient healthcare costs. It calculates the percentage of older workers who incur inpatient (*n* = 967) and outpatient (*n* = 6719) costs by type of work with 95% confidence interval (CI). It also computes the average inpatient and outpatient healthcare expenditure for formal and informal older workers and tests the significance of differences using *t*-test.

**Figure 2.**
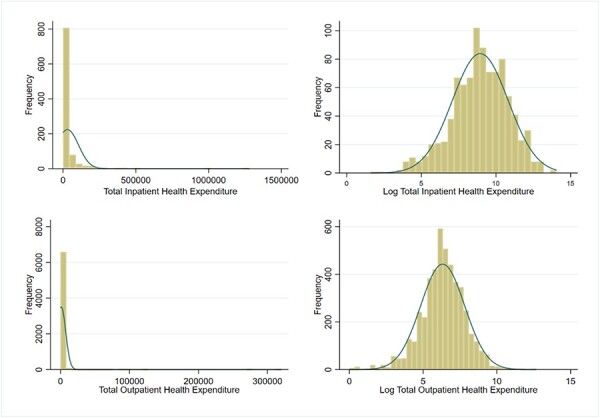
Distribution of healthcare expenditure (in INR) of working population (*n* = 7040)

The OOP health care payments for inpatient and outpatient are found to be extremely right skewed (see Figure 2) with many small values (including zeros) and few very large ones. This is a common feature of health care expenditure and cost data worldwide. Generally, the log-normal distribution and the gamma distribution are used to model positive health care expenditures, which are heavily right-skewed distributions. As observed from Figure 2, the log transformation of OOP payments follows a normal distribution. For this type of data, two-part model (TPM) is appropriate where positive health care expenditures are not normally distributed.

TPM uses both the property of gamma distribution and binomial distribution in order to deal with skewed information making it an appropriate statistical tool, which is supported by existing literatures (García, 2013; Humphreys, 2013; Deb and Norton, 2018). There are two components of TPM, the first component uses probit or logistic regression, where the dependent variable is in a binary form. This component provides a likelihood of incurring OOP payments on healthcare services. The second component either uses ordinary least squares (OLS) or generalized linear models (GLMs), which predicts the level of OOP payments for healthcare conditional on non-zero value.

As per the aforesaid information, the present study utilized a probit model for the first component, and a GLM with log transformation and gamma distribution for the second component. Here the dependent variables are inpatient and outpatient healthcare expenditures incurred in the last 12 months.

Following is the equation of TPM:

1. First part, a probit model can be written as:

(2) $$P\left( {Y > 0{\mathrm{|}}X} \right) = {\ }\phi \left( {X^{\prime}\beta } \right)\ldots\ldots\ldots..$$

Where,

- Y is OOP healthcare expenditure,
- X is the vector of independent variables,
- Φ is the cumulative distribution function of the standard normal distribution.

1. The second part, a GLM, is:

(3) $$E\left( {Y{\mathrm{|}}X,{\ }Y > 0} \right) = {g^{ - 1}}\left( {X^{\prime}\alpha } \right)\,\,\ldots\ldots\ldots..$$

Where,

- g^−1^ is the inverse of the link function,
- α is the vector of parameters to be estimated.

So, combining equation (2) and equation (3) gives the TPM which is:


(4) $$E\left( {Y{\mathrm{|}}X} \right) = {\ }\phi \left( {X^{\prime}\beta } \right)*{g^{ - 1}}\left( {X^{\prime}\alpha } \right)\,\,\ldots\ldots\ldots\ldots.$$

Calculation of OOP healthcare expenditure may not represent actual burden of expenses on individuals. A holistic approach is followed to estimate the burden of OOP health care expenditure which is described in equation (1). The outcome variable derived from this equation is graphically presented for each threshold of 10, 20 and 40%, respectively. The association between type of work and burden of OOP payments is assessed by applying chi-square test. Further, probit regression models are employed for each threshold, where the key exposure variable is ‘type of work’. Both, crude and adjusted models are estimated to examine the relationship between the type of work and the burden of OOP healthcare expenditure. All the analyses are performed in STATA software.

## Results

Table 1 exhibits the descriptive statistics of healthcare costs incurred by older workers in India, categorized by their type of work. Regarding healthcare costs, 8.4% of formal older workers incur inpatient expenses, compared to 7.3% of informal older workers. For outpatient costs, 56.0% of formal workers and 52.1% of informal workers incur these expenses.

**Table 1.** Distribution of older workers by type of work

Indicators	Formal	95% CI	Informal	95% CI
*Key exposure variable*				
*Type of healthcare cost*				
Inpatient cost incurred				
No	91.6	(90.8, 92.4)	92.7	(92.1, 93.2)
Yes	8.4	(7.6, 9.2)	7.3	(6.8, 7.9)
Outpatient cost incurred				
No	44.0	(42.6, 45.5)	47.9	(46.8, 49.0)
Yes	56.0	(54.5, 57.4)	52.1	(51.0, 53.2)
*Socio-economic and demographic indicates*				
Gender				
Male	80.0	(78.8, 81.1)	65.5	(64.4, 66.5)
Female	20.0	(18.9, 21.2)	34.5	(33.5, 35.6)
Age groups, years				
60–65	45.6	(44.2, 47.0)	55.5	(54.4, 56.6)
65+	54.4	(53.0, 55.8)	44.5	(43.4, 45.6)
Caste groups				
General	34.0	(32.6, 35.4)	20.0	(19.1, 20.9)
ST	14.7	(13.7, 15.8)	21.9	(21.0, 22.8)
SC	13.6	(12.7, 14.7)	18.9	(18.0, 19.7)
OBC	37.7	(36.3, 39.1)	39.3	(38.2, 40.3)
Religion				
Hindu	77.5	(76.2, 78.7)	73.7	(72.7, 74.6)
Muslim	10.0	(9.2, 10.9)	9.5	(8.9, 10.2)
Others	12.6	(11.6, 13.5)	16.8	(16.0, 17.6)
Education level				
Low	49.4	(48.0, 50.9)	82.4	(81.6, 83.2)
Middle	28.9	(27.6, 30.2)	13.9	(13.1, 14.7)
High	21.7	(20.5, 22.9)	3.7	(3.3, 4.1)
Marital status				
Currently married	79.6	(78.4, 80.7)	74.9	(73.9, 75.8)
Others	20.4	(19.3, 21.6)	25.1	(24.2, 26.1)
Residence				
Rural	55.5	(54.0, 56.9)	77.3	(76.4, 78.2)
Urban	44.6	(43.1, 46.0)	22.7	(21.8, 23.6)
Wealth				
Low	25.3	(24.1, 26.6)	40.7	(39.6, 41.8)
Middle	31.1	(29.7, 32.4)	35.1	(34.1, 36.1)
High	43.6	(42.2, 45.1)	24.2	(23.3, 25.2)
Household size				
1	3.4	(2.9, 4.0)	6.0	(5.5, 6.5)
2	21.3	(20.1, 22.5)	21.1	(20.3, 22.1)
3	12.0	(11.1, 13.0)	11.8	(11.1, 12.6)
4+	63.3	(61.9, 64.7)	61.1	(60.0, 62.1)
*Health*				
Health conditions				
No condition	36.8	(35.4, 38.2)	45.4	(44.3, 46.5)
CHC	40.9	(39.4, 42.3)	30.0	(29.0, 31.0)
Depression	9.0	(8.2, 9.8)	12.8	(12.0, 13.5)
CHC and depression	13.3	(12.4, 14.3)	11.8	(11.1, 12.6)
Childhood health				
Good/fair	97.0	(96.4, 97.4)	97.5	(97.2, 97.8)
Poor	3.0	(2.6, 3.6)	2.5	(2.2, 2.8)
Health insurance				
No	77.4	(76.1, 78.6)	82.0	(81.1, 82.8)
Yes	22.6	(21.4, 23.9)	18.0	(17.1, 18.8)
*Lifestyle behaviours*				
Smoking/consuming tobacco				
No	55.0	(53.5, 56.4)	49.7	(48.6, 50.8)
Yes	45.0	(43.6, 46.5)	50.3	(49.2, 51.4)
Drinking alcohol				
No	87.8	(86.8, 88.7)	83.7	(82.9, 84.5)
Yes	12.2	(11.3, 13.2)	16.3	(15.5, 17.1)
Vigorous activities				
Never	55.0	(53.5, 56.4)	44.3	(43.2, 45.4)
Rare	18.0	(16.9, 19.1)	20.7	(19.9, 21.6)
Everyday	27.0	(25.8, 28.4)	35.0	(33.9, 36.0)
Moderate activities				
Never	32.8	(31.4, 34.1)	29.9	(28.9, 30.9)
Rare	15.5	(14.5, 16.6)	15.2	(14.4, 16.0)
Everyday	51.7	(50.3, 53.2)	54.9	(53.8, 56.0)
Yoga/Pranayam				
Never	78.7	(77.5, 79.8)	86.7	(85.9, 87.4)
Rare	5.9	(5.2, 6.6)	4.0	(3.6, 4.5)
Everyday	15.5	(14.4, 16.5)	9.3	(8.7, 10.0)
*Regions*				
North	15.8	(14.8, 16.9)	13.8	(13.1, 14.6)
Central	15.5	(14.4, 16.5)	12.0	(11.3, 12.7)
East	19.1	(18.0, 20.3)	18.0	(17.2, 18.9)
Northeast	9.9	(9.0, 10.8)	15.5	(14.7, 16.3)
West	12.0	(11.1, 13.0)	10.5	(9.9, 11.2)
South	17.4	(16.4, 18.6)	22.9	(22.0, 23.9)
Union Territories	10.3	(9.4, 11.2)	7.3	(6.7, 7.8)
Overall	4575		7984	

The table also presents the distribution of older workers, highlighting various socio-economic, demographic, health and lifestyle characteristics. The majority of older workers are male, with 80% in the formal economic activity and 65.5% in the informal economic activity. In terms of caste groups, most formal older workers belong to the General (34%) and OBC (37.7%) categories, while the majority of the informal workers belong to the OBC group (39.3%). Approximately 82.4% of informal older workers have a low education level, compared to 49.4% of formal older workers. Around 43.6% of formal older workers come from a high wealth category, whereas most informal workers come from a low wealth category. Regarding health conditions, 40.9% of formal older workers have CHC compared to 30.0% of informal older workers. The percentage of smoking/consuming tobacco is 45.0% among formal workers and 50.3% among informal workers.

### OOP healthcare expenditure by type of work

**Figure 3.**
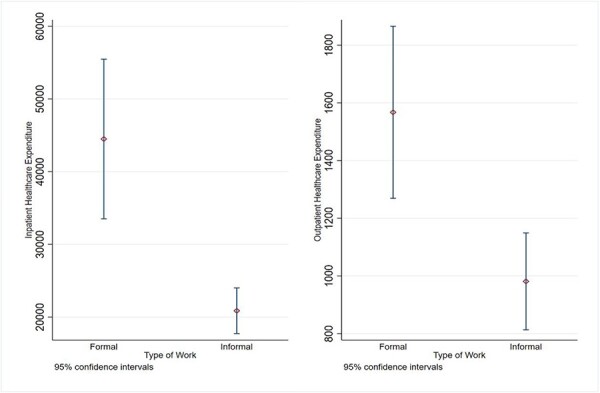
Average healthcare expenditure (in INR) by type of work

Figure 3 illustrates that the average inpatient healthcare costs among formal and informal older workers are INR 44 495 and INR 20 866 (*P* < 0.01), respectively. Moreover, the study finds that the average outpatient healthcare expenditure is significantly higher (*P* < 0.01) for formal older workers (INR 1567) than their informal (INR 981) counterparts.

Table 2 provides a comprehensive view of healthcare spending data in INR, broken down by various socio-economic and demographic factors. This spending is divided into inpatient and outpatient costs, which are further split into formal and informal older workers. It is observed that males generally incur higher healthcare costs than females across most categories. Among older workers, healthcare spending tends to rise with age, education and wealth. Older workers from the general and OBC caste groups have high inpatient healthcare costs, while those from the general and ST caste groups have high outpatient costs.

**Table 2.** Average healthcare expenditure (in INR) by type of health care and type of work

	Inpatient health expenditure			Outpatient health expenditure		
	Formal	Informal	Total	Formal	Informal	Total
Predictors	Mean (min, max)	Mean (min, max)	Mean (min, max)	Mean (min, max)	Mean (min, max)	Mean (min, max)
*Socio-economic* *and demographic indicates*						
Gender						
Male	47 744 (0–1 281 100)	23 133 (0–300 000)	33 559 (0–1 281 100)	1633 (0–276 000)	1042 (0–320 100)	1297 (0–320 100)
Female	31 954 (0–400 000)	15 266 (0–204 500)	20 604 (0–400 000)	1307 (0–85 000)	868 (0–30 600)	982 (0–85 000)
Age groups, years						
60–65	37 136 (0–440 000)	19 018 (0–237 000)	24 868 (0–440 000)	1554 (0–124 000)	849 (0–32 200)	1081 (0–124 000)
65+	48 911 (0–1 281 100)	22 853 (0–300 000)	34 857 (0–1 281 100)	1577 (0–276 000)	1138 (0–320 100)	1328 (0–320 100)
Caste groups						
General	53 387 (0–1 281 100)	24 717 (0–285 000)	39 694 (0–1 281 100)	2043 (0–276 000)	1001 (0–30 200)	1536 (0–276 000)
ST	27 488 (0–373 780)	20 272 (0–250 500)	22 350 (0–373 780)	2187 (0–100 000)	1321 (0–71 600)	1609 (0–100 000)
SC	25 915 (0–410 000)	14 671 (0–199 000)	18 516 (0–410 000)	820 (0–20 000)	962 (0–320 100)	921 (0–320 100)
OBC	47 019 (0–1 000 000)	21 619 (0–300 000)	31 051 (0–1 000 000)	1203 (0–100 000)	879 (0–40 000)	993 (0–100 000)
Religion						
Hindu	45 645 (0–1 281 100)	19 283 (0–300 000)	30 123 (0–1 281 100)	1512 (0–276 000)	803 (0–32 800)	1077 (0–276 000)
Muslim	31 871 (0–356 044)	25 723 (0–285 000)	27 841 (0–356 044)	1635 (0–100 000)	969 (0–40 000)	1218 (0–100 000)
Others	45 322 (0–400 000)	25 829 (0–250 500)	32 682 (0–400 000)	1937 (0–100 000)	2233 (0–320 100)	2129 (0–320 100)
Education level						
Low	28 533 (0–1 000 000)	18 499 (0–300 000)	21 449 (0–1 000 000)	1003 (0–100 000)	940 (0–320 100)	957 (0–320 100)
Middle	57 334 (0–1 281 100)	31 027 (0–250 500)	45 192 (0–1 281 100)	1723 (0–100 000)	1185 (0–32 200)	1487 (0–100 000)
High	65 550 (0–512 000)	23 622 (0–88 000)	56 533 (0–512 000)	2546 (0–276 000)	1100 (0–13 000)	2246 (0–276 000)
Marital status						
Currently married	45 193 (0–1 281 100)	23 088 (0–300 000)	31 884 (0–1 281 100)	1674 (0–276 000)	919 (0–70 000)	1218 (0–276 000)
Others	41 758 (0–1 000 000)	12 296 (0–140 000)	23 902 (0–1 000 000)	1166 (0–85 000)	1161 (0–170 000)	1163 (0–320 100)
Residence						
Rural	33 720 (0–1 000 000)	19 211 (0–300 000)	23 961 (0–1 000 000)	1576 (0–276 000)	907 (0–70 000)	1109 (0–276 000)
Urban	58 950 (0–1 281 100)	26 578 (0–285 000)	44 574 (0–1 281 100)	1557 (0–124 000)	1234 (0–170 000)	1413 (0–320 100)
Wealth						
Low	23 509 (0–373 780)	14 814 (0–285 000)	17 270 (0–373 780)	1173 (0–124 000)	850 (0–70 000)	940 (0–124 000)
Middle	43 426 (0–1 281 100)	20 253 (0–185 000)	28 961 (0–1 281 100)	1360 (0–100 000)	882 (0–49 000)	1044 (0–100 000)
High	55 243 (0–1 000 000)	29 484 (0–300 000)	42 813 (0–1 000 000)	1904 (0–276 000)	1305 (0–170 000)	1616 (0–320 100)
Household size						
1	25 891 (0–190 000)	15 291 (100–140 000)	19 670 (0–190 000)	616 (0–5000)	835 (0–30 200)	773 (0–30 200)
2	55 327 (0–1 281 100)	15 034 (0–182 800)	29 449 (0–1 281 100)	1216 (0–60 000)	1075 (0–71 600)	1129 (0–71 600)
3	41 325 (0–410 000)	17 922 (0–185 000)	27 110 (0–410 000)	2515 (0–124 000)	1090 (0–32 200)	1659 (0–124 000)
4+	42 748 (0–1 000 000)	24 746 (0–300 000)	32 130 (0–1 000 000)	1570 (0–276 000)	943 (0–320 100)	1183 (0–320 100)
*Health*						
Health conditions						
No condition	28 093 (0–512 000)	13 575 (0–119 400)	18 186 (0–512 000)	1527 (0–276 000)	786 (0–71 600)	1016 (0–276 000)
CHC	51 336 (0–1 281 100)	26 182 (0–300 000)	37 596 (0–1 281 100)	1704 (0–100 000)	1218 (0–320 100)	1440 (0–320 100)
Depression	13 144 (100–150 000)	11 381 (0–96 000)	11 954 (0–150 000)	637 (0–30 000)	621 (0–20 000)	626 (0–30 000)
CHC and depression	52 438 (0–440 000)	23 761 (0–237 000)	34 945 (0–440 000)	1599 (0–124 000)	1142 (0–40 000)	1318 (0–124 000)
Childhood health						
Good/fair	43 574 (0–1 281 100)	19 838 (0–300 000)	29 243 (0–1 281 100)	1560 (0–276 000)	985 (0–320 100)	1203 (0–320 100)
Poor	57 438 (0–400 000)	43 623 (320–199 000)	48 677 (0–400 000)	1583 (0–15 000)	904 (0–12 500)	1196 (0–15 000)
Health insurance						
No	41 796 (0–512 000)	22 281 (0–300 000)	30 121 (0–512 000)	1706 (0–276 000)	1037 (0–320 100)	1295 (0–320 100)
Yes	51 219 (0–1 281 100)	17 568 (0–204 500)	30 556 (0–1 281 100)	1118 (0–40 000)	811 (0–18 000)	924 (0–40 000)
*Lifestyle behaviours*						
Smoking/consuming tobacco						
No	46 238 (0–1 281 100)	22 267 (0–300 000)	32 477 (0–1 281 100)	1654 (0–124 000)	1236 (0–320 100)	1408 (0–320 100)
Yes	42 457 (0–1 000 000)	19 581 (0–285 000)	27 999 (0–1 000 000)	1470 (0–276 000)	762 (0–32 800)	1010 (0–276 000)
Drinking alcohol						
No	43 230 (0–1 281 100)	22 428 (0–300 000)	31 214 (0–1 281 100)	1611 (0–276 000)	991 (0–320 100)	1234 (0–320 100)
Yes	57 950 (0–1 000 000)	13 589 (0–182 800)	24 353 (0–1 000 000)	1211 (0–28 000)	927 (0–16 250)	1016 (0–28 000)
Vigorous activities						
Never	41 509 (0–440 000)	24 843 (0–300 000)	32 273 (0–440 000)	1782 (0–124 000)	997 (0–71 600)	1349 (0–124 000)
Rare	47 319 (101–1 281 100)	13 590 (0–140 000)	25 385 (0–1 281 100)	1067 (0–40 400)	881 (0–30 600)	943 (0–40 400)
Everyday	52 835 (0–1 000 000)	17 650 (0–285 000)	28 617 (0–1 000 000)	1452 (0–276 000)	1023 (0–320 100)	1157 (0–320 100)
Moderate activities						
Never	52 907 (0–1 281 100)	30 206 (0–300 000)	39 261 (0–1 281 100)	2095 (0–124 000)	1293 (0–320 100)	1621 (0–320 100)
Rare	28 538 (0–373 780)	18 851 (0–150 000)	22 368 (0–373 780)	1472 (0–49 000)	970 (0–71 600)	1163 (0–71 600)
Everyday	42 847 (0–1 000 000)	14 005 (0–250 500)	25 760 (0–1 000 000)	1312 (0–276 000)	845 (0–32 800)	1016 (0–276 000)
Yoga/Pranayam						
Never	39 816 (0–1 000 000)	20 629 (0–300 000)	27 721 (0–1 000 000)	1681 (0–276 000)	979 (0–320 100)	1229 (0–320 100)
Rare	50 394 (1700–280 000)	27 148 (80–125 000)	39 054 (80–280 000)	924 (0–12 000)	738 (0–15 700)	820 (0–15 700)
Everyday	65 630 (0–1 281 100)	20 616 (0–100 000)	46 278 (0–1 281 100)	1278 (0–20 000)	1103 (0–32 200)	1197 (0–32 200)
*Regions*						
North	52 158 (0–1 000 000)	25 268 (0–237 000)	38 156 (0–1 000 000)	1862 (0–276 000)	726 (0–15 700)	1165 (0–276 000)
Central	34 951 (50–440 000)	17 271 (0–81 750)	25 217 (0–440 000)	601 (0–40 000)	420 (0–10 570)	501 (0–40 000)
East	28 110 (0–194 000)	12 291 (0–178 975)	19 721 (0–194 000)	1670 (0–124 000)	655 (0–32 800)	1052 (0–124 000)
Northeast	49 407 (300–373 780)	20 294 (0–250 500)	29 904 (0–373 780)	3592 (0–57 800)	2106 (0–71 600)	2624 (0–71 600)
West	71 188 (0–1 281 100)	20 017 (0–181 565)	35 117 (0–1 281 100)	928 (0–15 900)	940 (0–32 200)	935 (0–32 200)
South	35 779 (0–410 000)	26 630 (0–300 000)	29 820 (0–410 000)	1326 (0–100 000)	1119 (0–40 000)	1182 (0–100 000)
Union Territories	55 551 (0–400 000)	11 488 (0–100 000)	30 857 (0–400 000)	2156 (0–100 000)	1770 (0–320 100)	1947 (0–320 100)
Overall	44 495 (0–1 281 100)	20 866 (0–300 000)	30 249 (0–1 281 100)	1567 (0–320 100)	981 (0–170 000)	1205 (0–320 100)

Note: CHC: Chronic Health Conditions.

In terms of religion, Hindu formal older workers have higher inpatient healthcare costs, whereas Muslim informal older workers have higher costs for the same. As expected, in urban areas, both informal and formal older workers have higher inpatient healthcare costs compared to their rural counterparts. However, outpatient costs are roughly the same for formal older workers in both urban and rural areas. Regarding health conditions, older workers with either CHC or both CHC and depression have higher healthcare costs. This trend is observed in both informal and formal workers.

Table 3 of this study provides the parameter estimates of the probit and OLS parts of the TPM. On the other hand, Table 4 presents the marginal estimates of average inpatient and outpatient healthcare expenditure by ‘type of work’ and ‘health conditions’. As far as the magnitude of OOP healthcare cost is concerned, there is an evident significant difference between the OOP healthcare cost of formal and informal workers. Specifically, older workers engaged in informal activities pay 0.380 (*P* < 0.05) and 0.107 (*P* < 0.001) units less for inpatient and outpatient expenditure, respectively. Moreover, the study observes that the average healthcare spending of informal older workers for inpatient care was INR 1113 (*P* < 0.01) and for outpatient care was INR 55 (*P* < 0.05) less compared to formal older workers. The study further reveals that older workers suffering from only CHC, as well as both CHC and depression, incur more inpatient and outpatient OOP healthcare expenses compared to those with no condition. The findings are found to be statistically significant at *P* < 0.001.

**Table 3.** Estimates from two-part models (probit and GLM): the effect of type of work on healthcare expenditure

	Inpatient healthcare expenditure		Out-patient healthcare expenditure	
Predictors	Probit (95% CI)	GLM (95% CI)	Probit (95% CI)	GLM (95% CI)
Type of work				
Formal®				
Informal	−0.051 (−0.129, 0.026)	−0.380* (−0.672, −0.088)	−0.029 (−0.083, 0.025)	−0.107*** (−0.197, −0.018)
*Socio-economic and demographic indicates*				
Gender				
Male®				
Female	−0.052 (−0.148, 0.044)	−0.119 (−0.498, 0.261)	0.174*** (0.11, 0.239)	0.080 (−0.027, 0.186)
Age groups, years				
60–65®				
65+	0.044 (−0.026, 0.115)	0.196 (−0.073, 0.465)	0.024 (−0.025, 0.072)	0.039 (−0.04, 0.119)
Caste groups				
General®				
ST	−0.165* (−0.299, −0.03)	0.2 (−0.381, 0.782)	−0.483** (−0.572, −0.393)	0.139 (−0.017, 0.294)
SC	−0.033 (−0.146, 0.081)	−0.388 (−0.816, 0.041)	−0.054 (−0.131, 0.023)	−0.248** (−0.369, −0.126)
OBC	0.015 (−0.077, 0.107)	0.1 (−0.242, 0.442)	−0.171** (−0.234, −0.107)	−0.038 (−0.138, 0.063)
Religion				
Hindu®				
Muslim	−0.073 (−0.195, 0.049)	−0.131 (−0.602, 0.339)	0.187*** (0.105, 0.269)	0.027 (−0.101, 0.154)
Others	−0.009 (−0.128, 0.111)	0.182 (−0.297, 0.660)	0.031 (−0.051, 0.114)	−0.193** (−0.33, −0.056)
Education level				
Low®				
Middle	0.025 (−0.07, 0.121)	0.258 (−0.086, 0.602)	−0.028 (−0.096, 0.039)	0.206*** (0.095, 0.316)
High	−0.145* (−0.283, −0.007)	0.420 (−0.109, 0.949)	0.03 (−0.062, 0.123)	0.268*** (0.118, 0.418)
Marital status				
Currently married®				
Others	−0.139** (−0.232, −0.046)	−0.129 (−0.495, 0.237)	0.000 (−0.061, 0.061)	−0.101* (−0.2, −0.002)
Residence				
Rural®				
Urban	−0.172*** (−0.258, −0.086)	0.344* (0.018, 0.670)	−0.072* (−0.131, −0.013)	−0.077 (−0.173, 0.019)
Wealth				
Low®				
Middle	−0.005 (−0.094, 0.084)	0.317 (−0.025, 0.658)	0.119*** (0.059, 0.18)	−0.051 (−0.153, 0.051)
High	−0.003 (−0.104, 0.099)	0.605** (0.215, 0.995)	0.155*** (0.085, 0.224)	0.027 (−0.089, 0.143)
Household size	−0.034 (−0.072, 0.005)	0.031 (−0.116, 0.178)	−0.011 (−0.038, 0.015)	0.003 (−0.041, 0.047)
*Health*				
Health conditions				
No condition®				
CHC	0.435*** (0.350, 0.520)	0.600** (0.261, 0.938)	0.452*** (0.396, 0.508)	0.301*** (0.209, 0.393)
Depression	0.160* (0.033, 0.286)	0.074 (−0.458, 0.606)	−0.150*** (−0.232, −0.067)	−0.001 (−0.156, 0.155)
CHC and depression	0.557*** (0.452, 0.663)	0.556** (0.153, 0.960)	0.312*** (0.236, 0.388)	0.324*** (0.201, 0.448)
Childhood health				
Good/fair®				
Poor	0.217* (0.031, 0.402)	0.636 (−0.008, 1.280)	0.127 (−0.015, 0.27)	0.191 (−0.03, 0.413)
Health insurance				
No®				
Yes	0.098* (0.018, 0.178)	−0.090 (−0.394, 0.214)	0.054 (−0.002, 0.11)	−0.073 (−0.165, 0.019)
*Lifestyle behaviours*				
Smoking/consuming tobacco				
No®				
Yes	0.078* (0.001, 0.155)	0.019 (−0.280, 0.318)	0.181*** (0.129, 0.234)	−0.097*** (−0.183, −0.01)
Drinking alcohol				
No®				
Yes	−0.085 (−0.19, 0.019)	−0.295 (−0.706, 0.117)	0.054 (−0.017, 0.124)	0.034 (−0.085, 0.153)
Vigorous activities				
Never®				
Rare	−0.107* (−0.206, −0.009)	0.057 (−0.326, 0.441)	0.132*** (0.064, 0.199)	0.007 (−0.103, 0.117)
Everyday	−0.178*** (−0.266, −0.090)	0.327 (−0.017, 0.670)	0.103** (0.044, 0.162)	−0.031 (−0.128, 0.067)
Moderate activities				
Never®				
Rare	0.036 (−0.072, 0.144)	−0.359 (−0.745, 0.027)	0.094* (0.017, 0.171)	−0.146* (−0.275, −0.017)
Everyday	−0.028 (−0.114, 0.057)	−0.411* (−0.741, −0.082)	0.150* (0.090, 0.211)	−0.248* (−0.349, −0.147)
Yoga/Pranayam				
Never®				
Rare	−0.061 (−0.228, 0.106)	0.264 (−0.359, 0.887)	0.02 (−0.09, 0.13)	−0.127 (−0.304, 0.05)
Everyday	−0.034 (−0.147, 0.079)	0.207 (−0.231, 0.646)	0.057 (−0.019, 0.132)	0.062 (−0.058, 0.182)
*Regions*				
North®				
Central	−0.201* (−0.340, −0.063)	−0.053 (−0.613, 0.506)	−0.489** (−0.584, −0.394)	0.012 (−0.159, 0.184)
East	−0.257** (−0.382, −0.132)	−0.467 (−0.936, 0.002)	0.164** (0.081, 0.247)	0.117 (−0.01, 0.245)
Northeast	−0.066 (−0.215, 0.084)	−0.326 (−0.919, 0.268)	−0.211* (−0.313, −0.108)	0.582* (0.410, 0.754)
West	−0.003 (−0.135, 0.129)	0.058 (−0.438, 0.553)	0.105* (0.013, 0.198)	0.027 (−0.12, 0.173)
South	−0.034 (−0.153, 0.085)	−0.051 (−0.457, 0.355)	−0.055 (−0.139, 0.029)	0.413** (0.276, 0.55)
Union Territories	−0.042 (−0.196, 0.113)	−0.391 (−0.964, 0.182)	−0.436* (−0.546, −0.325)	0.094 (−0.107, 0.295)
Constant	−1.300 (−1.547, −1.054)	9.647 (8.621, 10.673)	−0.759 (−0.948, −0.571)	6.871 (6.56, 7.182)

® reference category;

*** (*P* < 0.001);

** (*P* < 0.01);

* (*P* < 0.05).

**Table 4.** Estimates from two-part regression models (probit and GLM): MEs of healthcare expenditure by type of work across inpatient and outpatient healthcare for older workers

	Average healthcare expenditure	
Predictors	Inpatient (95% CI)	Outpatient (95% CI)
Type of work		
Formal®		
Informal	−1113** (−1925, −301)	−55* (−96, −13)
Health		
No conditions®		
CHC	2585*** (1811, 3358)	296*** (252, 342)
Depression	396 (−272, 1065)	−43 (−87, 0.1)
CHC and depression	3184*** (1876, 4493)	243*** (178, 308)

® reference category;

*** (*P* < 0.001),

** (*P* < 0.01),

* (*P* < 0.05);

marginal estimate holding all other variables in the model at their means, all the costs are in Indian Rupee (INR).

Apart from aforementioned findings, Table 3 shows that physical activity plays a significant role in reducing inpatient OOP healthcare expenses for older workers who perform moderate activities every day. In the case of urban India, the inpatient OOP healthcare expenses were higher than in rural areas. Additionally, older workers belonging to SC population and those performing moderate activities incur less outpatient OOP healthcare expenses. Conversely, education level had a direct relationship with outpatient OOP health care costs, as the expenses escalate with the increase in education level among older workers.

### Burden of OOP payments by type of work

**Figure 4.**
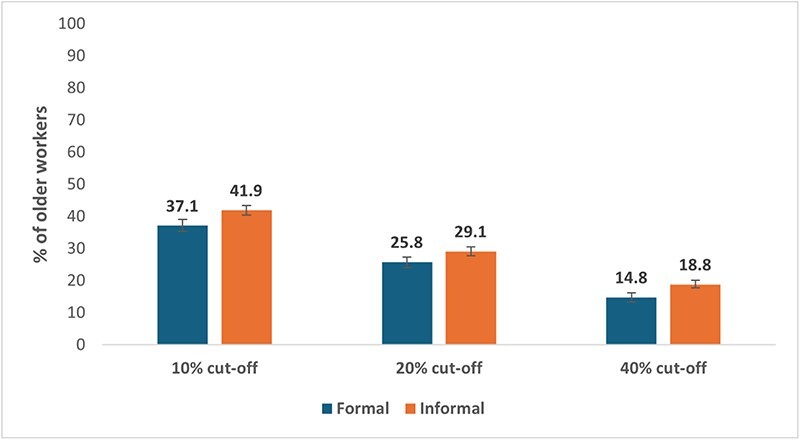
Percentage of older workers experiencing catastrophic healthcare payments

The burden of OOP healthcare payments is significantly (*P*<0.001) higher for informal older workers when their payments exceed a certain proportion of their income (Figure 4). For example, the CHE estimates based on the predefined thresholds of 10, 20 and 40% indicate that the percentage of informal older workers facing catastrophic payments are 41.9% (95% CI: 0.40, 0.43), 29.1% (95% CI: 0.28, 0.31) and 18.8% (95% CI: 0.18, 0.20), respectively.

Supplementary Appendix Table A1 reveals that the burden of catastrophic payments is notably high among older female workers. Specifically, 45.5% are affected at a 10% cut-off, 32.8% at a 20% cut-off and 21.2% at a 40% cut-off. Additionally, among various caste groups, STs experience a significant burden of catastrophic payments, with incidences of 45.5% at a 10% cut-off, 32.2% at a 20% cut-off and 21.2% at a 40% cut-off. When examining the burden of catastrophic payments across religions, Hindus show a higher incidence at the 20% and 40% cut-offs.

Older workers with low and middle education levels face the highest burden of catastrophic payments across all cut-offs compared to their highly educated counterparts. In terms of health conditions, those suffering from CHC have a high incidence (41.9% at 10% cut-off, 29.9% at 20% and 40% cut-offs), and the incidence is even higher for those suffering from both CHC and depression (46.2% at 10% cut-off, 33.8% at 20% and 40% cut-offs). Regionally, the burden of catastrophic payments among older workers is high in the Eastern and Southern regions across all cut-offs.

Table 5 presents the results of a probit regression model and marginal effect (hereafter ME) estimates for catastrophic payments. The dependent variable is the catastrophic payments, with three different cut-off points: 10, 20 and 40%. For each model, both unadjusted and adjusted coefficients are provided. The results are statistically significant and robust to the inclusion of control variables. For instance, in model-1 (10% cut-off), the adjusted coefficient for informal older workers is 0.092 (*P* < 0.01), implying a higher probability of facing catastrophic health expenditure for these workers than for formal workers. The ME estimates indicate that about 41.1% (*P* < 0.001) of informal older workers incur CHE at the 10% threshold. Similarly, in model-3 (40% cut-off), the adjusted coefficient suggests that informal older workers have a greater probability (*β* = 0.128, *P* < 0.01; ME: 0.177) of experiencing catastrophic health expenditure than formal older workers (ME: 0.146). In all models, the Akaike Information Criterion (AIC) decreases after adjustment, indicating that the adjusted models provide a better fit to the data.

**Table 5.** Probit regression model estimates: Marginal effect estimates of catastrophic healthcare payments by type of work

Models	β coefficient (95% CI)	Marginal effects (95% CI)	AIC
**Model 1: Dep variable= Catastrophic payments (cut-off 10%)**			
Unadjusted: Type of Work			
Formal®		0.371*** (0.352, 0.389)	9430.591
Informal	0.124*** (0.026, 0.185)	0.418*** (0.404, 0.433)	
Adjusted: Type of Work			
Formal®		0.376*** (0.356, 0.396)	9152.535
Informal	0.092** (0.024, 0.161)	0.411*** (0.396, 0.427)	
**Model 2: Dep variable= Catastrophic payments (cut-off 20%)**			
Unadjusted: Type of Work			
Formal®		0.257*** (0.241, 0.274)	8289.871
Informal	0.101** (0.036, 0.166)	0.291*** (0.278, 0.305)	
Adjusted: Type of Work			
Formal®		0.256*** (0.239, 0.274)	8006.945
Informal	0.076* (0.004, 0.148)	0.282*** (0.268, 0.296)	
**Model-3: Dep variable= Catastrophic payments (cut-off 40%)**			
Unadjusted: Type of Work			
Formal®		0.148*** (0.134, 0.161)	6444.121
Informal	0.160** (0.088, 0.233)	0.188*** (0.176, 0.199)	
Adjusted: Type of Work			
Formal®		0.146*** (0.131, 0.160)	6243.312
Informal	0.128** (0.046, 0.209)	0.177*** (0.165, 0.189)	

® reference category;

*** (p<0.001),

** (p<0.01),

* (p<0.05);

Adjusted model controlled for all other covariates; marginal estimates hold all other variables in the models at their means.

## Discussion

India’s economy is among the fastest growing in the world. However, health is not one of the primary focal points in this ambitious development story. In reality, some of the low and lower-middle income countries, including India, appear better in contrast due to their disproportionately tight healthcare budgets. A meagre public health spending has resulted in poor risk protection, with households bearing the brunt of healthcare expenditures (Rao, 2016; Sharma, 2024). For instance, according to the National Health Accounts (2019–20) data released in 2023, 52% of healthcare expenses in India are paid for out of pocket, making it one of the highest rates of OOP expenditure in the world. While public health spending accounts for 1/35 of GDP (Sharma, 2024). The informal sector is a key feature of any developing economy, and India is no exception. Each year, a significant portion of the workforce engages in informal work. The Indian social security system, which dates back to 1947, offers minimal benefits to these informal workers, raising concerns about productivity in the Indian economy (Majumdar and Borbora, 2013). The country has one of the largest economic-adjusted dependency ratios (Rani, Goli and Reddy, 2023). Informal workers often receive low wages and lack formal contracts, paid leave, health benefits or social security (Ramana Murthy, 2019) which leads to a vicious cycle of poverty and volatile livelihoods, thereby forcing workers to remain engaged even beyond the retirement age of 60 years.

Previous research has indicated that informal older workers often suffer from poor physical and mental health issues (Chowdhury *et al*., 2023). Over 80% of the older population either totally or partially dependent on others for their financial stability due to the unpredictable nature of much of the job they conduct and the low compensation (Goli *et al*., 2019). Additionally, existing programs and policies have been insufficient in providing financial protection due to inadequate coverage and benefits, a lack of awareness and an over-reliance on private facilities. Consequently, examining the burden of OOP health payments on these workers, in comparison to their formal counterparts is vitally important.

The findings of this study illuminate the above-mentioned issue, revealing that informal older workers, on an average, pay INR 1113 less for inpatient care and INR 55 less for outpatient care compared to older formal workers (in absolute terms). However, despite lower OOP payments among informal workers, the burden of these payments is quite high relatively. For instance, 18% of them spend more than 40% of their income on healthcare expenditure compared to 15% of formal older workers. A similar trend is observed for the 20% (28% vs 26%) and 10% (41% vs 38%) thresholds. Apparently, a substantial wage gap between formal and informal workers is a primary factor in this situation (see Supplementary Appendix Figure A2). Recent studies have also highlighted that the average daily wage of an informal worker is significantly lower than that of a formal worker (Mishra, 2017; Ramana Murthy, 2019; Kumar and Pandey, 2021). This disparity forms a considerable barrier to accessing health services, particularly private healthcare facilities and inpatient services. Furthermore, the fear of wage loss contributes to lower OOP payments among older informal workers, leading to untreated illnesses, a common occurrence among the impoverished older population in India (Mukherjee and Karmakar, 2008; Pandey *et al*., 2017; Srivastava and Gill, 2020). Also, financial limitations increase the burden of OOP payments among older informal workers compared to their formal counterparts. Previous research has extensively documented that socio-economically disadvantaged households with at least one older member bear a relatively higher burden of OOP expenses (Mohanty *et al*., 2014; 2023; Pandey, 2017; Pandey *et al*., 2018a; Sahoo *et al*., 2021). Older formal workers with higher incomes and a greater capacity to pay often have financial protection, either directly or indirectly, through pensions and provident funds (Sakthivel and Pinaki, 2006) as well as the means to afford private healthcare insurance. Conversely, older informal workers face financial challenges due to their lower daily wage, lack of financial protection and absence of healthcare insurance.

In a highly informal economies like India, most of the older population is not covered under any financial protection schemes (Jeyashree *et al*., 2018; Agrawal and Mishra, 2021; Sahoo *et al*., 2021). The majority of the programs have failed to provide minimum financial protection to the older population, especially those belonging to the BPL category and the informal sector (Gokhale, 2003; Bharati and Singh, 2013; Dhillon and Ladusingh, 2013). This has been attributed to implementation issues, lack of education and a general lack of awareness among the population (Karan *et al*., 2017; Agrawal and Mishra, 2021; Prinja *et al*., 2022; Srivastava *et al*., 2023).

The escalating risk of CHC, the cost of treatment and an over-reliance on the private healthcare sector are the primary factors contributing to the significant burden of OOP payments among the older population (Brinda *et al*., 2015; 2016; Anand, 2016; Srivastava and Gill, 2020; Sahoo *et al*., 2021; Mohanty *et al*., 2023; Puri and Singh, 2022). The current study underscores that older workers afflicted with either CHC or a combination of CHC and depression are more prone to the burden of OOP payments.

Public and private health insurance schemes are typically the main mechanisms to manage OOP payments and their associated burdens. However, in a country like India, only a small fraction of the older population is covered under these health insurance schemes (Jeyashree *et al*., 2018; Agrawal and Mishra, 2021). There have been certain health insurance programs at the state or central level in India, with RSBY and Pradhan Mantri Jan Arogya Yojana (PMJAY) being two significant initiatives aimed at providing universal health coverage. Yet, both programs have encountered challenges related to implementation and coverage (Narayana, 2010; Ghosh, 2014; Srivastava *et al*., 2023). A study by Karan *et al*. (2017) points out that RSBY did not offer significant protection for impoverished households due to delayed reimbursements to hospitals and a low coverage limit (INR 30 000) under the scheme. In contrast, PMJAY offers coverage is 17 times larger (INR 0.5 million per family) and aimed to provide protection to poorest 40% of the households (PMJAY, 2018). It is indicated in a study by Mohanty *et al*. (2023) that the initiation of PMJAY has increased the health insurance coverage and reduced geographical and socio-economic inequalities. However, other research implied that this scheme has not succeeded in alleviating the burden of OOP expenditure (Garg *et al*., 2020). A study conducted in Southern India revealed that extensive coverage does not necessarily equate to financial protection (Garg *et al*., 2019). The study by Trivedi *et al*. (2022) identifies awareness of benefits and eligibility as the principal problems with PMJAY and RSBY. It indicates that most people learn about these programs through informal sources like friends, relatives and newspapers. In addition to PMJAY’s awareness issues, other areas needing improvement include the functionality of the helpdesk and investment in capacity building for Ayushman Mitra (Trivedi *et al*., 2022). Furthermore, India still suffers from low literacy levels, inadequate knowledge of healthcare access and severe social hierarchies coupled with social discrimination and a highly hierarchal healthcare delivery system (Brinda *et al*., 2016; Rao, 2016; Acharya, 2018; Pandey *et al*., 2018a; Prinja *et al*., 2022), which creates a significant supply and demand gap in the access of social security programs (Agrawal and Mishra, 2021).

While other studies have explored the benefits of health insurance schemes on healthcare utilization, the results regarding their impact on reducing OOP payments or providing financial protection remain inconclusive (Karan *et al*., 2017; Prinja *et al*., 2017; Srivastava *et al*., 2023). Garg *et al*. (2020) further state that despite PMJAY’s robust mechanism for cashless service, which covers both pre- and post-operative care and restricts hospitals from overcharging, this mechanism does not prevent private hospitals from overcharging patients. Therefore, to strengthen PMJAY and ensure it provides financial protection to the poor and informal older workers, the aforementioned issues need to be addressed.

This study has some limitations that should be acknowledged. The research relied on cross-sectional data from a single wave, due to the absence of nationally representative longitudinal data for India’s older population. This limitation prevented the study from establishing a causal relationship between the type of work, OOP payments and the burden of these payments. Although the study’s focus on inpatient and outpatient healthcare costs by type of work allowed for a preliminary analysis with a somewhat adequate sample size, a larger sample would be required for an in-depth analysis. This could potentially facilitate a more nuanced understanding of healthcare expenditure by type of work in India. Furthermore, there is a lack of national or international research on this topic, making it challenging to corroborate the current findings. Nevertheless, the study used research on the conditions of informal workers, OOP payments and catastrophic health expenditure of the older population to substantiate its results. As such, this research is unique in providing new insights into the situation of informal older workers in India, which could assist policymakers in formulating better health policies to allocate resources and services for these vulnerable population segments.

In conclusion, India must find creative ways to overcome the social welfare, economic and health issues it faces in order to meet the growing financial shocks experienced by older informal workers. Enhancing insurance coverage and offering high-quality, subsidized public health facilities are pivotal in improving healthcare accessibility and protecting the older population from financial catastrophe. Furthermore, addressing the health concerns of older people, including financial catastrophes, necessitates the implementation of health insurance and other security systems, as well as the promotion of active, healthy and productive ageing. Also, the World Health Organization also advocates for developing countries to enhance healthcare equity and affordability by implementing Universal Health Insurance, aiming to ensure everyone has access to medical services without suffering financial hardship. In addition, future policies and regulations for informal workers should also consider their difficulties and livelihood challenges by strengthening a social security system/financial provision. This could be accomplished through raising awareness about *the programs and the* rights of informal workers using information and communication technology. The use of such technology is indispensable for superior reporting and fostering health literacy. A geriatric care-friendly healthcare system, particularly in the lower tiers of the system (i.e. sub-centres, primary health centres, community health centres or newly established health and wellness centres), can prevent avoidable healthcare spending and help develop a healthy and active older population in the country. Furthermore, monitoring the progress of these policies is essential as it is a fundamental component of the global sustainable development agenda. Altogether, universal health coverage and financial protection schemes for informal older workers will shield them from the burden of OOP expenses or other economic repercussions which are the main reasons for their later life work engagement.

## Supplementary Material

czae074_Supp

## Data Availability

The data and procedure to access the data underlying this article will be shared upon reasonable request to the corresponding author. The authors affirm that the data are accessible to others following the same procedure, and they had no special access rights.

## Abbreviations

AIC: Akaike Information Criterion, ABP: Ayushman Bharat Program, BPL: Below Poverty Line, CES-D: Centre for Epidemiological Studies-Depression, CHC: Chronic Health Conditions, CHE: Catastrophic Healthcare Expenditure, CI: Confidence Interval, GLM: Generalized Linear Model, IIPS: International Institute for Population Sciences, GDP: Gross Domestic Product, GOI: Government of India, ILO: International Labour Organization, INR: Indian Rupee, LASI: Longitudinal Ageing Study in India, ME: Marginal Effect, NCO: National Classification of Occupation, NSSO: National Sample Survey Organization, OBC: Other Backward Class, OLS: Ordinary Least Square, OOP: Out-of-pocket, PMJAY: Pradhan Mantri Jan Arogya Yojana, RSBY: Rastriya Swasthya Bima Yojana, SC: Scheduled Caste, ST: Scheduled Tribe, TPM: Two-Part Model

## Reflexivity statement

P.C. brings to the table their proficiency in the fields of ageing, health, and social science research. This research forms a part of the Ph.D. thesis. S.G., with a keen interest in population dynamics and its impact on economic development, family demography and gender and nutritional disparities, has significantly contributed to the conceptualization of this research. His extensive knowledge in the field of health economics has been invaluable. Both authors, who hail from India, have brought their unique perspectives and expertise to this research.
