# Supplementary material for: Informal employment and high burden of out-of-pocket healthcare payments among older workers: evidence from the Longitudinal Ageing Study in India
Source: Health Policy Plan. 2024 Aug 3;40(2):123–39. doi: 10.1093/heapol/czae074 (PMC11800986; doi:10.1093/heapol/czae074)
Supplement: czae074_Supp [file czae074_supp.zip › Supplementary_file_31July.docx]

**Appendix**

**Table A1: Proportion of older workers experiencing catastrophic healthcare payments by various indicator**

| **Indicators** | **Catastrophic healthcare payments cut-offs** | | |
| --- | --- | --- | --- |
|  | **10%** | **20%** | **40%** |
| **Type of work** |  |  |  |
| Formal | 37.1 | 25.8 | 14.8 |
| Informal | 41.9 | 29.1 | 18.8 |
| **Gender** |  |  |  |
| Male | 37.9 | 25.8 | 15.7 |
| Female | 45.5 | 32.8 | 21.2 |
| **Age groups** |  |  |  |
| 60-65 | 40.1 | 27.4 | 16.7 |
| 65+ | 40.0 | 28.3 | 17.9 |
| **Caste groups** |  |  |  |
| General | 42.2 | 29.1 | 17.3 |
| Scheduled Tribe | 45.5 | 32.2 | 21.2 |
| Scheduled Caste | 35.6 | 23.4 | 14.1 |
| Other Backward Class | 39.1 | 27.7 | 17.5 |
| **Religion** |  |  |  |
| Hindu | 39.2 | 27.4 | 17.0 |
| Muslim | 40.4 | 25.9 | 15.1 |
| Others | 46.1 | 33.4 | 21.9 |
| **Education Level** |  |  |  |
| Low | 41.2 | 28.6 | 17.8 |
| Middle | 40.3 | 29.1 | 17.6 |
| High | 32.4 | 20.9 | 13.7 |
| **Marital Status** |  |  |  |
| Currently married | 40.5 | 28.1 | 17.3 |
| Others | 38.7 | 27.0 | 17.2 |
| **Residence** |  |  |  |
| Rural | 42.6 | 29.9 | 18.8 |
| Urban | 34.5 | 23.3 | 14.0 |
| **Wealth** |  |  |  |
| Low | 41.3 | 28.3 | 18.0 |
| Middle | 40.0 | 28.4 | 17.2 |
| High | 39.1 | 26.9 | 16.8 |
| **Household Size** |  |  |  |
| 1 | 42.5 | 31.1 | 18.5 |
| 2 | 41.1 | 29.5 | 18.2 |
| 3 | 40.5 | 28.9 | 19.0 |
| 4+ | 39.5 | 26.8 | 16.6 |
| ***Health*** |  |  |  |
| **Health Conditions** |  |  |  |
| No condition | 37.2 | 24.8 | 24.8 |
| CHC | 41.9 | 29.9 | 29.9 |
| Depression | 32.3 | 20.0 | 20.0 |
| CHC and Depression | 46.2 | 33.8 | 33.8 |
| **Childhood Health** |  |  |  |
| Good/Fair | 39.8 | 27.6 | 27.6 |
| Poor | 47.5 | 33.3 | 33.3 |
| **Health Insurance** |  |  |  |
| No | 39.1 | 27.5 | 17.4 |
| Yes | 43.1 | 29.0 | 17.0 |
| ***Lifestyle behaviours*** |  |  |  |
| **Smoking/Consuming Tobacco** |  |  |  |
| No | 40.6 | 28.9 | 18.6 |
| Yes | 39.6 | 26.9 | 16.0 |
| **Drinking Alcohol** |  |  |  |
| No | 40.1 | 28.0 | 17.4 |
| Yes | 40.1 | 27.0 | 16.9 |
| **Vigorous activities** |  |  |  |
| Never | 40.3 | 28.4 | 17.8 |
| Rare | 41.4 | 30.1 | 18.2 |
| Everyday | 38.9 | 25.6 | 15.9 |
| **Moderate Activities** |  |  |  |
| Never | 42.6 | 30.4 | 19.6 |
| Rare | 39.3 | 27.9 | 16.3 |
| Everyday | 39.1 | 26.6 | 16.4 |
| **Yoga/Pranayam** |  |  |  |
| Never | 40.4 | 28.2 | 17.3 |
| Rare | 31.7 | 20.1 | 14.1 |
| Everyday | 41.7 | 28.7 | 18.7 |
| ***Regions*** |  |  |  |
| North | 34.0 | 23.1 | 13.4 |
| Central | 26.8 | 18.1 | 11.7 |
| East | 43.2 | 28.8 | 17.3 |
| Northeast | 64.4 | 47.6 | 31.1 |
| West | 43.9 | 28.8 | 16.9 |
| South | 43.7 | 32.1 | 20.5 |
| Union Territories | 24.1 | 16.7 | 11.3 |
| **Overall** | **40.09** | **27.86** | **17.3** |

**Figure-A1: % of poor households**


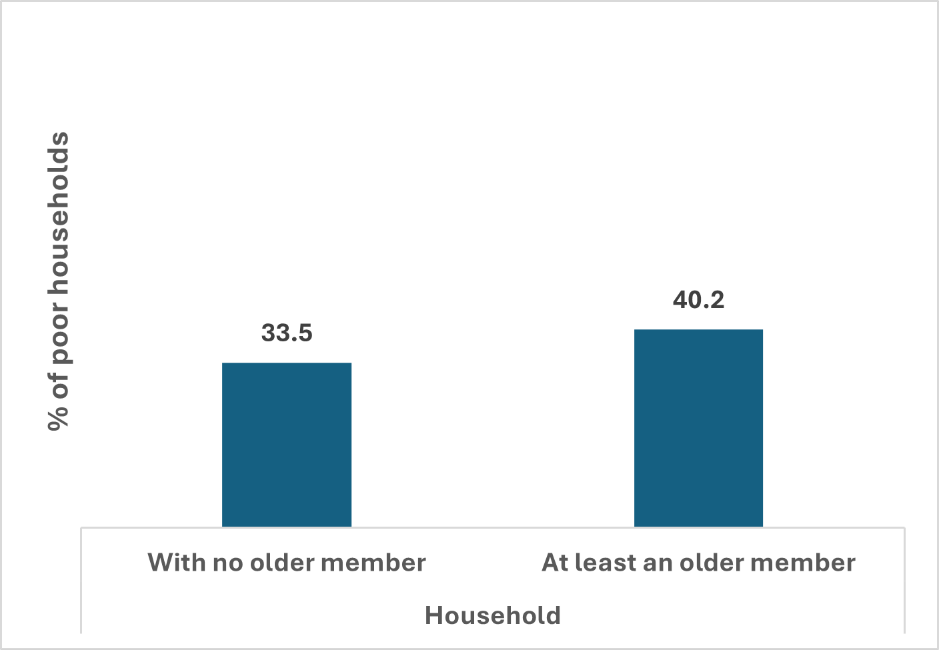


**Figure-A2: Monthly wages by type of work**


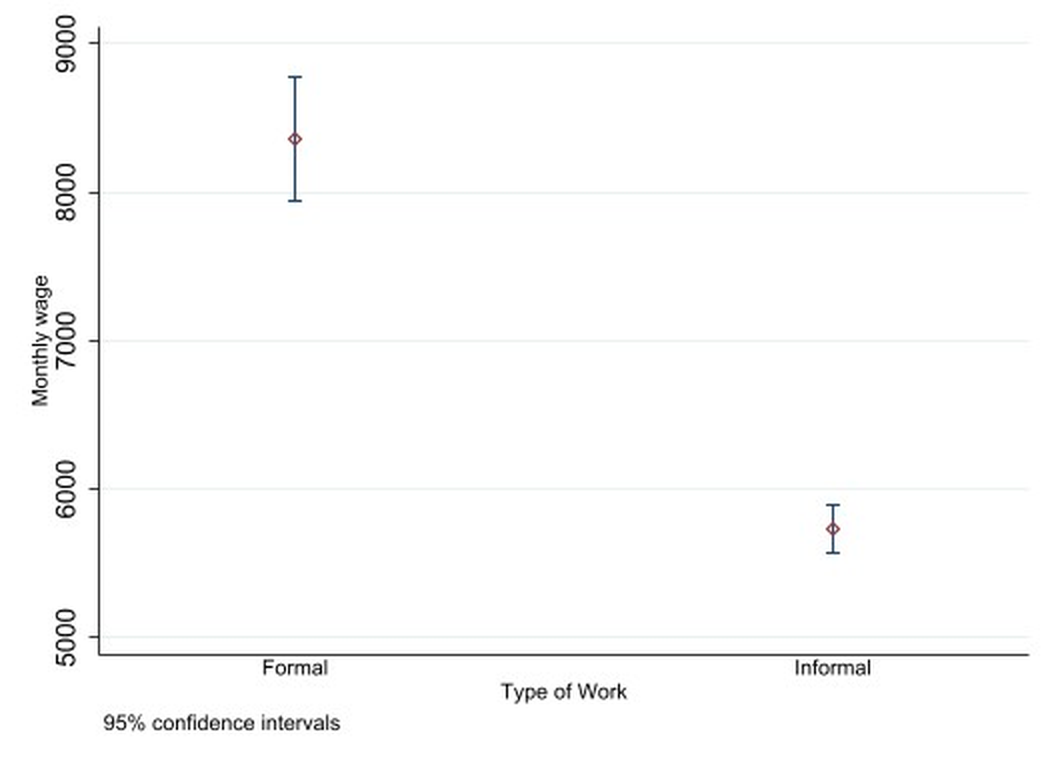


**Table-A2: Coverage of social insurance by type of work**

| **Social Insurances** | **Formal workers** | **Informal workers** | **Overall** |
| --- | --- | --- | --- |
| **Work Related Pension** | 6.2 | 0.0 | 3.1 |
| **Provident fund** | 5.0 | 0.0 | 2.5 |
| **Company Health insurance (not LIC)** | 1.9 | 1.2 | 1.4 |
| **Medical re-imbursement from employer** | 1.5 | 0.6 | 0.8 |
| **Worker’s employment insurance** | 0.6 | 0.0 | 0.4 |
| **Worker’s injury insurance** | 0.7 | 0.1 | 0.4 |
| **Total older workers** | **2,762** | **7,984** | **10,746** |

**Table-A3: Blinder-Oaxaca decomposition results**

|  | **Exp (b)** | **95% Confidence Interval** | |
| --- | --- | --- | --- |
| **Formal older workers-wage** | 601.04 | 550.37 | 656.39 |
| **Informal older workers-wage** | 521.79 | 494.42 | 550.68 |
| **Difference** | 1.159** | 1.041 | 1.277 |
| **Explained** | 1.071** | 1.018 | 1.124 |
| **Unexplained** | 1.080 | 0.983 | 1.177 |

Note: **(p<0.01)

**Figure-A3: Contribution of explained (7% of total gap) factors (Oaxaca decomposition)**


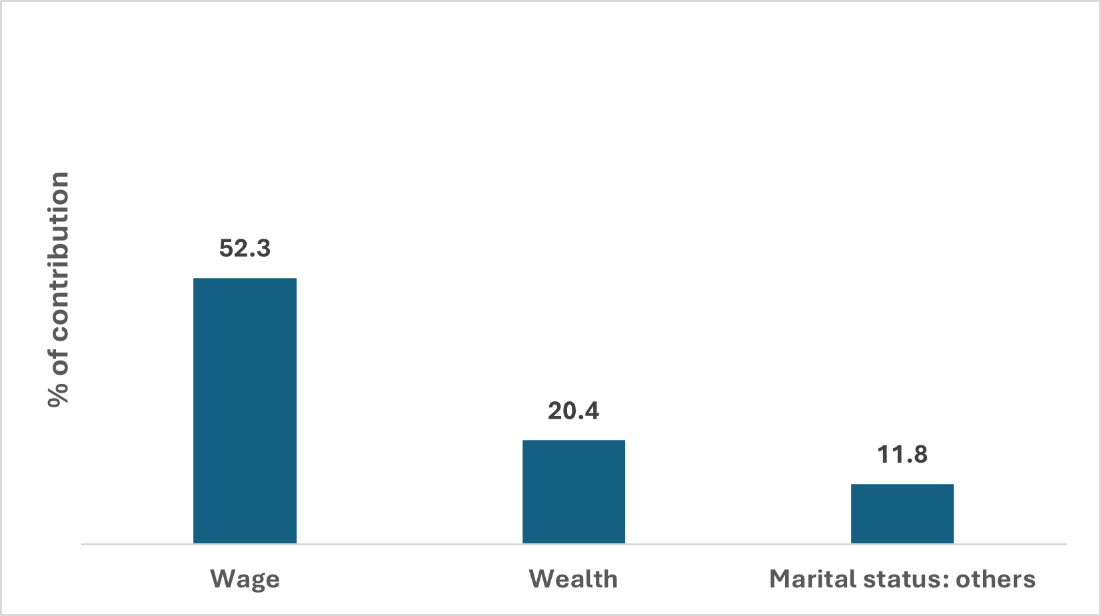


Note: Only factors with p<0.001 are presented in the figure
